# Effects of thermomechanical parameters on surface texture in filament materials extrusion: outlook and trends

**DOI:** 10.12688/f1000research.144965.1

**Published:** 2024-09-10

**Authors:** John D. Kechagias

**Affiliations:** 1 University of Thessaly, Karditsa, 43100, Greece

**Keywords:** material; extrusion; parameters; surface; texture; roughness;

## Abstract

The material extrusion process has been widely used to manufacture custom products. However, the surface texture varies due to the additive mechanism of the process, which depends on the layer height and surface orientation, resulting in varying average surface roughness values for inclined, flat and vertical surfaces. Different strand welding conditions result in non-uniform internal stresses, surface distortions, layer traces, weak bonding, non-uniform pores and material overflow. This paper comprehensively examines material extrusion process achievements in surface texture quality and studies and summarises the most influential processing parameters. Parameter effects are critically discussed for each topic; flat, inclined, and vertical surfaces. The results of this research help reduce post-processing.

## Introduction

**Figure 1.**
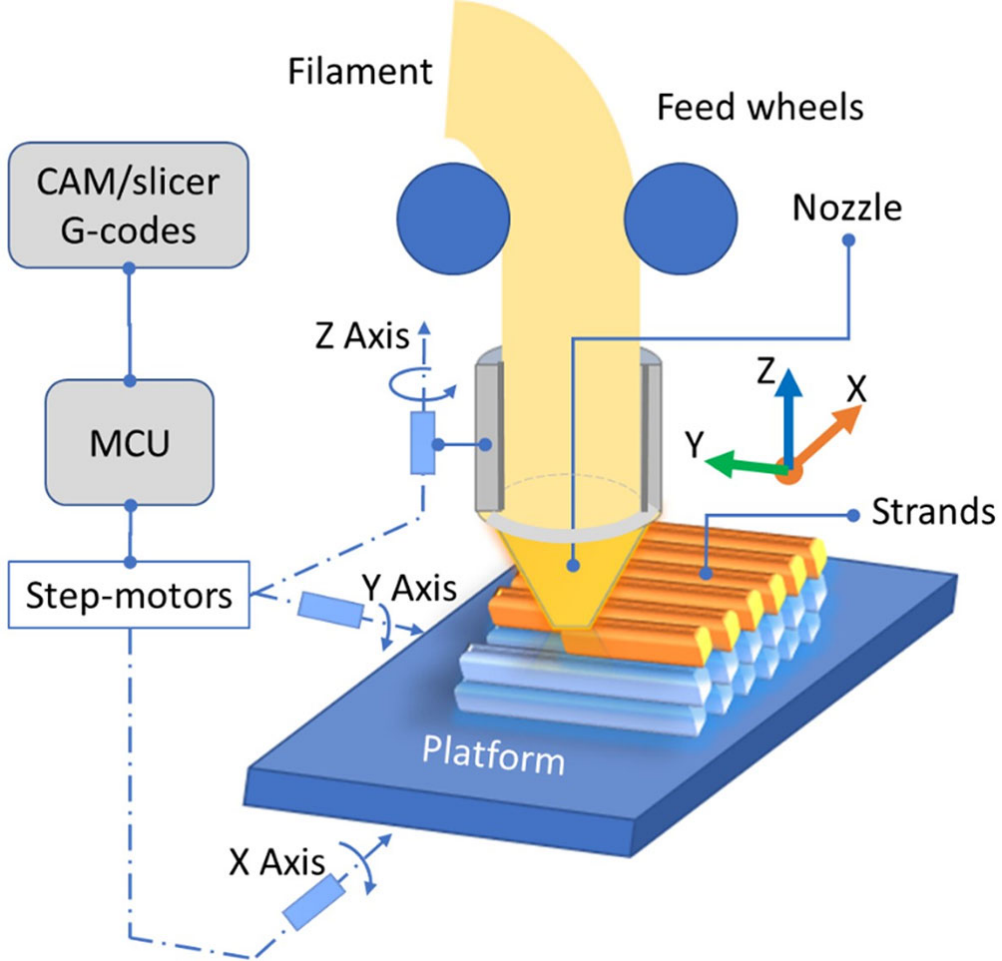
Essential components of the material extrusion process. **Source:** Author’s work.

The selective deposition of thermoplastic material by hot extrusion in sequentially patterned slices (layers) using computerised numerical control positioning is broadly known as the filament material extrusion (ME) process. ^1^^,^^2^ ME is a fully controlled thermomechanical process. ^3^^,^^4^ It consists of the following elements: (i) a machine control unit (MCU: micro-computer) which reads the g-code technological program, ^5^^,^^6^ (ii) three step-motors with the belts for the motion requirements in X, Y and Z axes, ^7^^,^^8^ (iii) the extruder mechanism (head carriage: filament, feed wheels, nozzle, fans, etc.), ^9^^,^^10^ and (iv) the bed in which the 3D-printed parts are created (see Figure 1). ^11^^,^^12^ The slicing (CAM) software handles the orientation of the STL model, ^13^ the slicing parameters, ^14^ and the selection of the infill structure and finally generates the g-codes. ^15^ ME technology is also known as fused filament fabrication (FFF), ^16^ fused deposition modeling (FDM) or material extrusion (ME). ^17^^,^^18^

Due to the cost-effectiveness of the ME process, ^19^ the timely manner and the customized orientation of the product, ^20^ ME 3D printed parts have been tested in many applications in the automotive, ^21^^,^^22^ aerospace, ^23^^,^^24^ electrical motors, ^25^ microwave absorption applications,, ^26^ electronics, ^27^^,^^28^ maritime, ^29^ medical, ^30^^,^^31^ neurosurgery, ^32^ orthotics, ^33^^,^^34^ dental, ^35^ antifouling, ^36^ heritage, ^37^^,^^38^ jewellery, ^39^ textile and fashion, ^40^^,^^41^ forensic, ^42^ and other sustainability and recyclability issues. ^43^ Surface texture (ST) parameters play an important role in surface engineering and are very important in determining the mechanical properties of ME 3D printed parts. ^44^^,^^45^ For example, higher surface roughness values of flat dogbone surfaces reduce the mechanical properties of the elements. ^46^ The inferior ST is a characteristic of all additive manufacturing techniques, compared with machining processes, ^47^ and post-processing methodologies have been proposed in the literature. ^48^^,^^49^ Therefore due to the progressive material deposition, additive manufacturing parts need post-processing with machining, abrasives, chemicals or cold spray coatings. ^50^^,^^51^

Since the ME 3D printing process has gained tremendous popularity due to its simplicity, ^52^^,^^53^ extensive materials, ^54^ organic infills, ^55^^,^^56^ fiber infills, ^57^^,^^58^ environmental friendliness, ^59^^,^^60^ and low budget, ^61^^,^^62^ several researchers have reviewed its achievements in material and strength issues, ^63^^,^^64^ benchmarking issues, ^65^^,^^66^ shape accuracy, ^67^^,^^68^ laser post-processing, ^69^^,^^70^ machining post-processing, ^71^ small-sized reliefs, ^72^^,^^73^ dimensional accuracy, ^74^ surface hardness, ^75^ friction and wear issues, ^76^ stair-stepping effect, ^77^^,^^78^ and flat inclined, horizontal and vertical surface roughness issues. Table 1 summarises and concludes the literature’s influential parameter investigations regarding the ME surface texture cases (sloped, flat and vertical) ^79^^–^^97^ between the end of 2000 and the start of 2023.

**Table 1.** Multi-parameter experimental studies of ME surface texture.

Researchers	Description	ME parameters studied	Conclusions
Mahapatra & Sood, 2012 ^79^	ABS P400 Prismatic part. Top, Bottom and side measurements ^*^Ra: 0.3 to 11.8μm	^**^H: 0.12, 0.17, 0.25 θ: 0, 15, 30 RA: 0, 30, 60 W: 0.4, 0.45, 0.5 AG: 0, 0.004, 0.008	Most influential parameters: Top: W, H, O Bottom: θ, RA, AG Side: H
Boschetto *et al.*, 2013 ^80^	ABS/ABSPlus/PC/ULTEM Inclined Ra > 15μm Rt > 75μm	H: varieties θ: 0-30	A neural network to predict Ra and Rt values was trained and suggested.
Chaidas *et al.,* 2016 ^81^	PLA Vertical surfaces of a thin-walled cuboid part. Ra, Rz, Rt, Rsm	T: 210, 220, 230 H=0.2	The increase in T decreases all roughness metrics. Ra between 14-17μm. Rz beteen 63-84μm. Rt between 68-124μm
Kim *et al.*, 2018 ^82^	PLA Top flat surfaces 0.4: 0.8 mm inside/outer nozzle tip diameter 1.38 μm Ra after optimisation	Variables H, S and Q	By adjusting the H, Q and S can achieve Ra values of about 1.38 μm.
Vyavahare *et al.*, 2020 ^95^	ABS Parts with pyramidal and conical features Ra: 9-38μm	H: 0.1-0.3 S: 30-90 θ: 0, 90, 180 WT: 0.8-1.4 T: 230-250 temperature.	H was the most critical parameter, followed by θ.
Aslani *et al.*, 2020 ^83^	PLA Vertical surfaces of a thin-walled cuboid part. Ra: 12-22μm Rz, Rt: 60-150μm Rsm: 196-321μm	T: 210, 220, 230 Wall Thickness: 1, 2, 3	The increase in T decreases all roughness metrics.
Biglete *et al.*, 2020 ^84^	ABS Vertical surfaces Ra: 5-30μm Rt: 80-150μm	H: 0.2, 0.3, 0.4 T: 220, 230, 240 S: 40,50,60	H dominates T moderate S not significant
Buj-Corral *et al.*, 2021 ^85^	PLA PLA Curved surfaces (hemispherical cups) Ra: 7-23 μm	T:195, 200, 205 H: 0.1, 0.2, 0.3 S: 30, 40, 50 EM: 0.4, 0.5, 0.6 ND: 93, 95, 97	H and ND were the most influential parameters.
Chaidas and Kechagias, 2022 ^86^	PLA wood Vertical surfaces Ra: 13-24μm Rt: 81-132 μm	T: 180, 190, 200, 210, 220 H: 0.1, 0.2, 0.3	Lower H values decreased the Ra values significantly. On the other hand, wood flour increases the Ra values considerably compared with pure PLA.
Caputo *et al.*, 2022 ^87^	PLA Top surface ironing effect on surface texture and mechanical dynamic response	S: 50,75, 100 T: 170, 195, 220 H: 0.12, 0.2, 0.28 ID: 5, 50, 100	Surface height variates between 0.326 mm and minus -0.32mm. Ra varieties between 0.8206 μm and 6.5526 μm. T was the most influential parameter for Ra.
Fountas *et al.*, 2022 ^93^	PLA Vertical surfaces Ra: 4.6-6.9μm	T: 210, 220, 230 Shells: 2, 3 ID: 10, 15, 20 Pattern: Diamond, Hexagonal, linear 0.2mm H (constant)	T and Pattern type were the most influential parameters.
Selvam *et al.*, 2022 ^88^	ABS Thin-walled parts Top and Bottom Ra: 2.5-5μm Inside vertical Ra: 1.4-1.85μm	S: 60, 105, 150 H: 0.1, 0.2, 0.3 T: 220, 232.5, 245	Vertical surfaces: S and H affected the Ra values significantly, while T was unimportant. Top/Bottom surfaces: All parameters significantly affect the Ra values.
Rakshit *et al.*, 2022 ^89^	PLA Inclined surfaces	RA and θ as variables	They achieved the lowest 5.3μm Ra value at 9 ^o^ θ and 0 ^o^ RA.
Rajesh *et al.* 2022 ^97^	ABS Flat surfaces of dogbone specimens Ra: 26-38μm	H: 0.15-0.25 T: 235-245 PlaT: 95-110	T was the most influential parameter, followed by H.
Vinoth Babu *et al.*, 2022 ^92^	CF/PLA composites Top surfaces of dogbones samples Ra>28μm	ID: 20, 40, 60, 80 H: 0.08, 0.25, 0.64 infill pattern: (a) rectilinear, (b) triangular and (c) hexagonal	The samples were excessively rough due to the carbon fibers.
Spahiu *et al.*, 2023 ^90^	PLA Top surfaces Ra: 2.4-5.5μm	T: 210, 220, 230 H: 0.15, 0.2, 0.25 S: 40, 50, 60	S was the most influential parameter. T was not significant. Lowering T values, the Ra improve slightly.
Bruijn *et al.*, 2023 ^91^	PEI Ultem™ 9085 Vertical bars	H: 0.254 ±45 lines style ID: 100 T: 185	They measured Ra values before annealing at about 17.3 and after annealing at 1.2μm and Rz values at about 70.4 and 6.3μm, accordingly.
Francis *et al.,* 2023 ^96^	ABS Flat surfaces of dogbones	H: 0.25 T: 230 PlaT: 110 ID: 100	They have improved surface texture and hardness with chemical and thermal treatments. They reduced the Ra to 0.819mμ (from 9μm) with 50s immersion time on acetone solvent NC1.
Kechagias & Zaoutsos, 2023 ^94^	PLA Dental implants	H: 0.2-0.16 ID: 0, 35, 70 PlatT: 50, 55, 60 T: 210, 220, 230	Optimising Ra and hardness of a dental implant (achieved Ra lower than 10μm).

* Ra (μm) is the arithmetic mean of the absolute values; Rz (μm) is the mean value of the sum of the highest and the deepest profile deviations; Rt (μm) is the difference between the highest peak and the deepest valley; Rsm (μm) the mean value of the width of the profile elements.

** Layer Height (H, mm); Orientation (θ, deg); Printing Speed (S, mm/s); Printing Temperature (T, ^o^C); Flow speed (Q, mm/s); Raster angle (RA, deg); Raster width (W, mm); Air gap (AG, mm); Infill density (ID, %); Nozzle Diameter (ND, mm); Extrusion Multiplier (EM, %); Platform Temperature (PlatT); Wall thickness (WT, mm).

The reader can be seen the variability of the measured mean and max height of the ME part’s surface texture (Ra and Rt). Ra values between 1-50μm and Rt between 50-150μm have been reported showing how important parameter tuning was in the ME process.

Analytically, for upper surfaces of dogbone parts, Ra values were between 0.8206 μm and 6.5526 μm for pure PLA, ^87^ whereas for CF/PLA composites measured Ra>28μm. ^92^ More, vertical Ra values were between 0.3 to 11.8μm for ABS P400 parts, ^79^ where for incleaned ABS/ABSPlus/PC/ULTEM parts measured Ra values higher than 15μm and Rt higher than 75μm. ^80^

It is noted that none of these studies summarises the surface texture in terms of inclined, vertical and flat surfaces as presented here. The surface texture of ME parts depends entirely on the arrangement of the processing parameters (perimeter, infill and top/bottom), ^98^^,^^99^ which in turn are affected by ‘filament properties’, ^100^ desired ‘strand welding’, ^75^ the ‘stair-stepping’ effect, ^101^ and part details. ^102^

**Figure 2.**
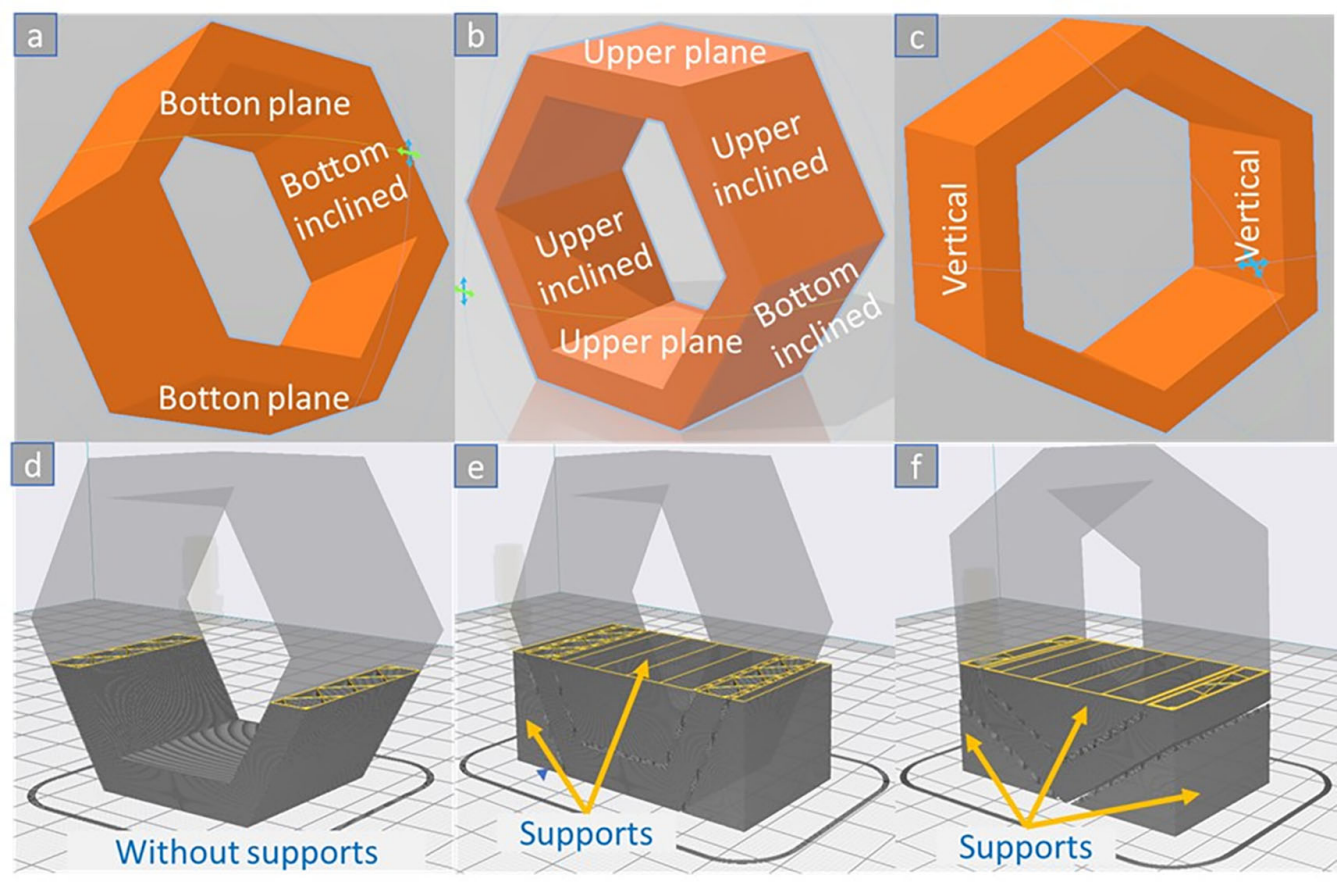
Vertical-built alternatives for ME 3D printing of a hexagonal ring: (a-c) types of surfaces and (d-f) types of build alternatives with and without supports. **Source:** Author’s work.

Figure 2 uses a ring-type hexagonal part to illustrate all cases of surface texture types engaged in the ME 3D printing process (the 3D model was designed in ‘3D builder’ and slice in ‘Creality v4.8.2’). In summary, we encounter flat, vertical or inclined surfaces. In addition, the inclined or flat top and bottom surfaces can meet without or be covered by a support structure, depending on the end user’s choice. ^103^^,^^104^

**Figure 3.**
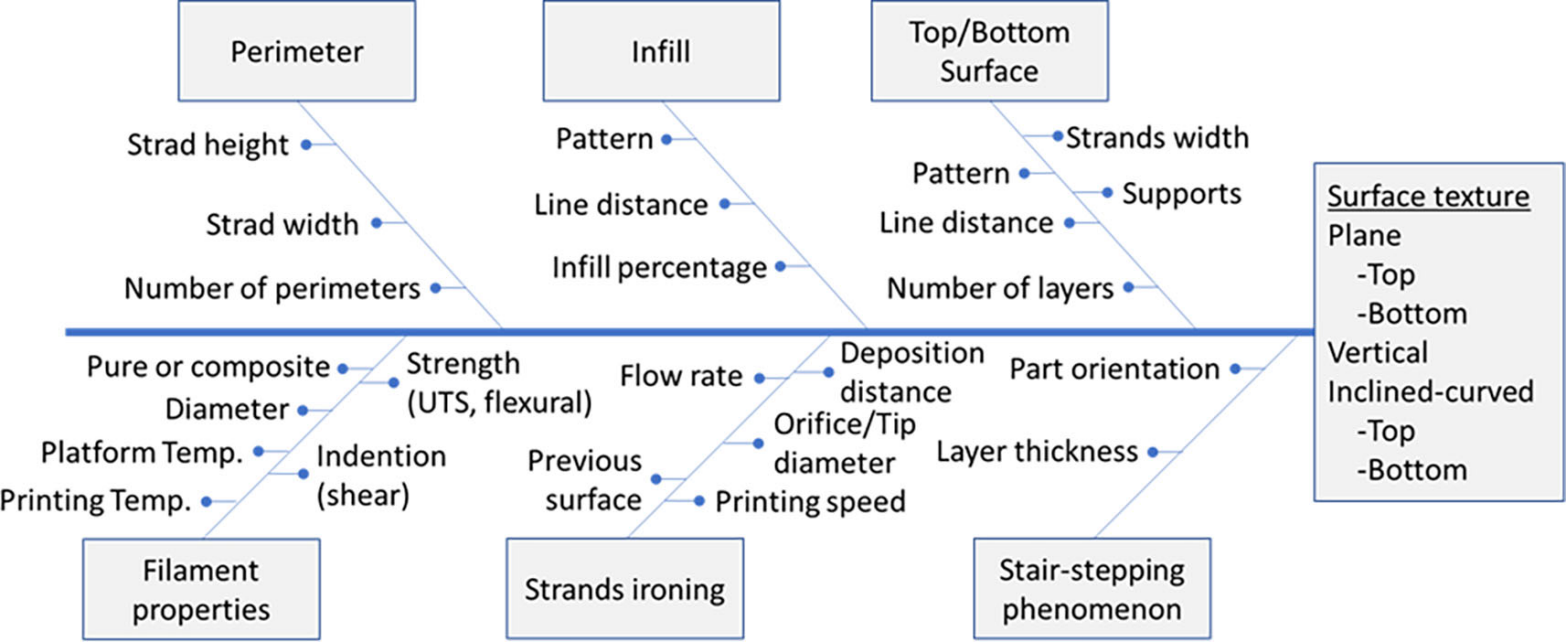
Surface texture affecting factors. **Source:** Author’s work.

Figure 3 illustrates all factors affecting ME surface texture using a cause-effect diagram. The texture of the perimeter, infill and top/bottom surfaces should be optimised according to filament properties, welding and staircase conditions. ^105^^,^^106^ It is noted here that some 3D printing processing parameters affect the perimeter, infill, and texture of the top/bottom surface differently. ^107^ For example, the width of the strand is not so important for vertical surfaces, but necessary to adjust the texture of the flat surface (top and bottom). Therefore, the quality and quantity of flat surfaces can affect the strand width choice, affecting the part’s time, energy consumption and mechanical strength. ^108^

Unfortunately, a comprehensive examination and detailed study on the surface texture of the ME 3D printing process in the context defined in Figure 3 has not yet existed in the literature. This study simultaneously considers the parameters’ effects on the material extrusion process’s surface texture regarding flat, vertical, and inclined surfaces. Though material type, component orientation, thermomechanical parameters, and total weld area affect surface types differently, this work shows the importance of optimizing the material extrusion process for different materials and component orientations, which is not emphasized in the existing literature. In addition, it summarises the most influential analytical and experimental work that has been done to study welding, stair-stepping, and filament effects, focusing on inclined vertical and planar (top and bottom) surface texture. Last, it communicates surface texturing issues and prospects for further study by the 3D research community. The results can help achieve a uniform surface texture and reduce post-processing and anisotropy of material extrusion parts.

## Methods

**Figure 4.**
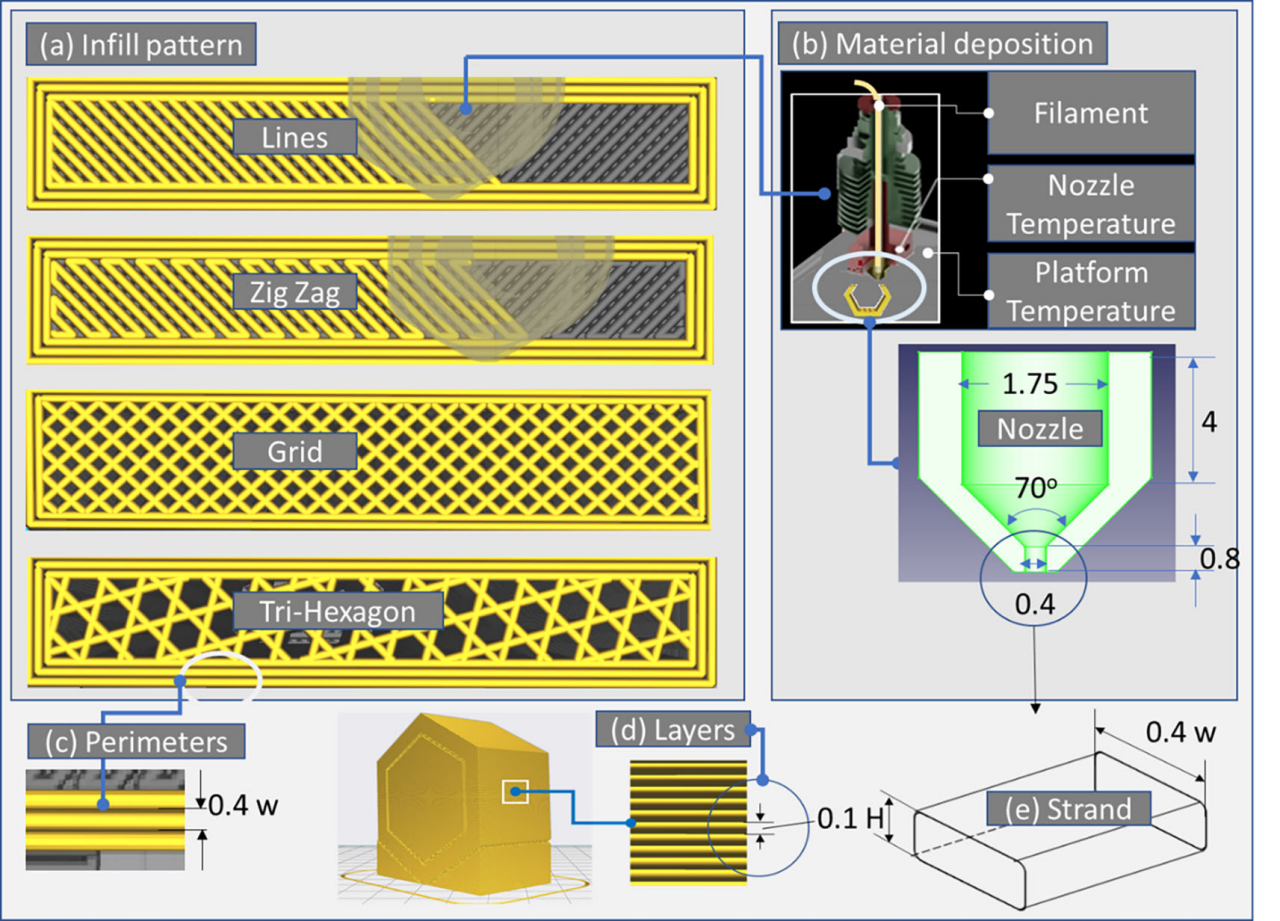
Explanation of the ME process selective material deposition procedure: (a) infill types, (b) nozzle characteristics, perimeter number, and (d) layers deposition. **Source:** Author’s work.

Each part manufactured by the ME process contains successive flat layers. Separately layer includes one or more perimeter lines and a defined infill structure according to the pattern selected by the end user (see Figure 4). ^109^ Infill follows the same configuration (pattern) along the Z-axis except for the flat top and bottom layers. ^110^^,^^111^ The user can select zero, one or more top and bottom layers. ^112^ In addition, support lines are created to support inclined sections or “island” type geometry. ^113^

Infill structures are deposited within the perimeter of each layer following several different deposition routes, e.g. lines, ^114^ zigzag, ^115^ grid, ^116^ and tri-hexagon, ^117^ to name a few ( Figure 4a). The infill density determines the final part weight (porosity), ^118^^,^^119^ while the pattern deposition angle determines the durability of the infill structure (static and dynamic mechanical response in the X and Y fabrication direction). ^120^^,^^121^ Different infill styles have been invented to reduce component weight and achieve controlled mechanical strength, ^122^ see gyroid infill structure. ^123^^,^^124^ Last but not least, layer thickness, filament properties (base and filler material), ^125^ nozzle inner and outer tip diameter (see Figure 4b), ^126^ and infill density determine the mechanical strength in the Z direction, as it has been shown in the literature that a lower layer thickness is beneficial due to a reduction in the porosity of the component (see Figure 4c-d). ^127^

### Flat surfaces

**Figure 5.**
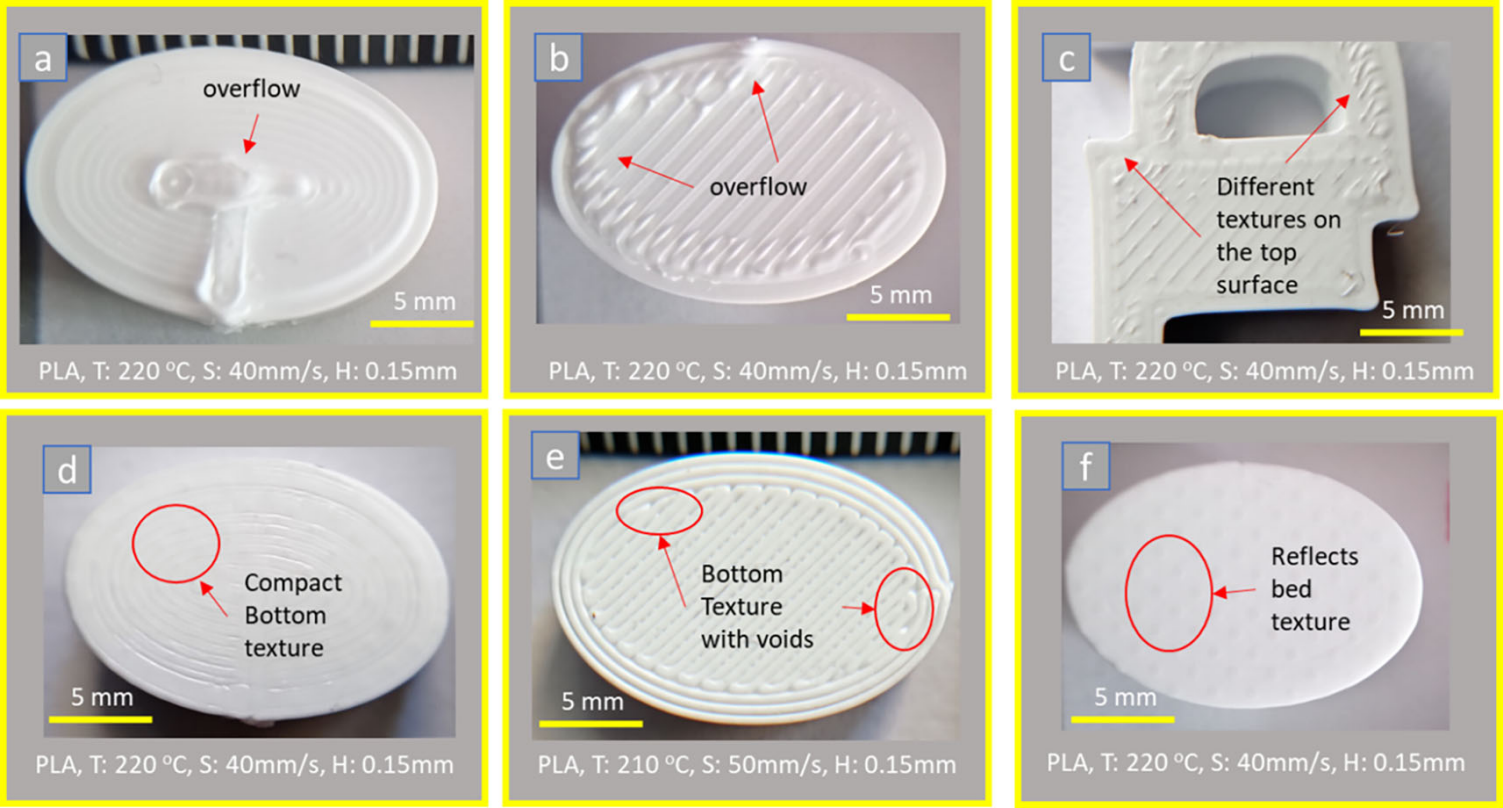
Flat surfaces with 100% flow rate: (a) concentric type of the upper surface, (b, c) line type of the upper surfaces, (d) concentric type of the bottom surface, and (e, f) line type of bottom surfaces with different deposition conditions. **Source:** Author’s work.

The top and bottom surfaces are characterized by 100% infill because they should be solid and allow no gaps between the perimeters and infill lines. ^96^ The flow speed (Q, mm/s), also known as flow rate (Fl, %), should be adjusted in cases of gaps or overflow material on top and bottom surfaces. Therefore an upper surface is affected by welding conditions the most, i.e., the material flow, strand width, deposition temperature, and printing speed; see Figure 5a-c. On the other hand, the bottom surface reflects the texture of the deposited surface and is also affected by the flow conditions of the material and the platform Z-leveling calibration; see Figure 5d-f. ^128^^,^^129^ Figure 5 shows the top and bottom surfaces of PLA parts 3D printed with different welding conditions; printing temperature (T), deposition speed (S) and strand height (H). Again, variable surface texture quality is evident, even in the same sample case ( Figure 5c). ^130^ Last but not least, in Figure 5a, the outer diameter of the nozzle is formed on the top surface due to the concentric filling (from the outside to the inside).

### Vertical and inclined surfaces

**Figure 6.**
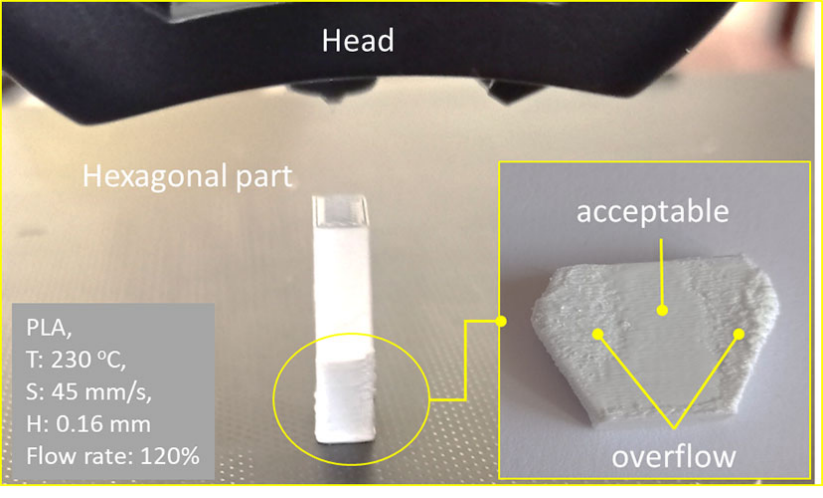
Overflow problems in vertical and inclined surfaces. **Source:** Author’s work.

Vertical and inclined surfaces primarily depend on the perimeters’ welding quality and on the infill characteristics. Bonding of the perimeters, infill lines, and successive layers of slices is achieved by applying thermal cycles between lines and layers, ^131^ which are directly affected by material properties, ^132^^,^^133^ processing parameters, ^134^^,^^135^ part size, ^136^ and orientation within the build bed. ^137^^,^^138^ Therefore, the nozzle, platform and vat temperature must be adjusted appropriately and according to the part volume and orientation. ^139^^,^^140^ Figure 6 illustrates the overflow problems during the ME process on different surfaces of the same part and with the same processing parameters. It is evident that some surface textures exhibit excellent quality and others are unacceptable due to material overflow. ^141^

## Results

This section reviews the above surface texture case studies according to the factors influencing deposition and orientation, e.g. the stair-stepping, overflow, material properties, and layer trace cases.

### Stair-stepping phenomenon

**Figure 7.**
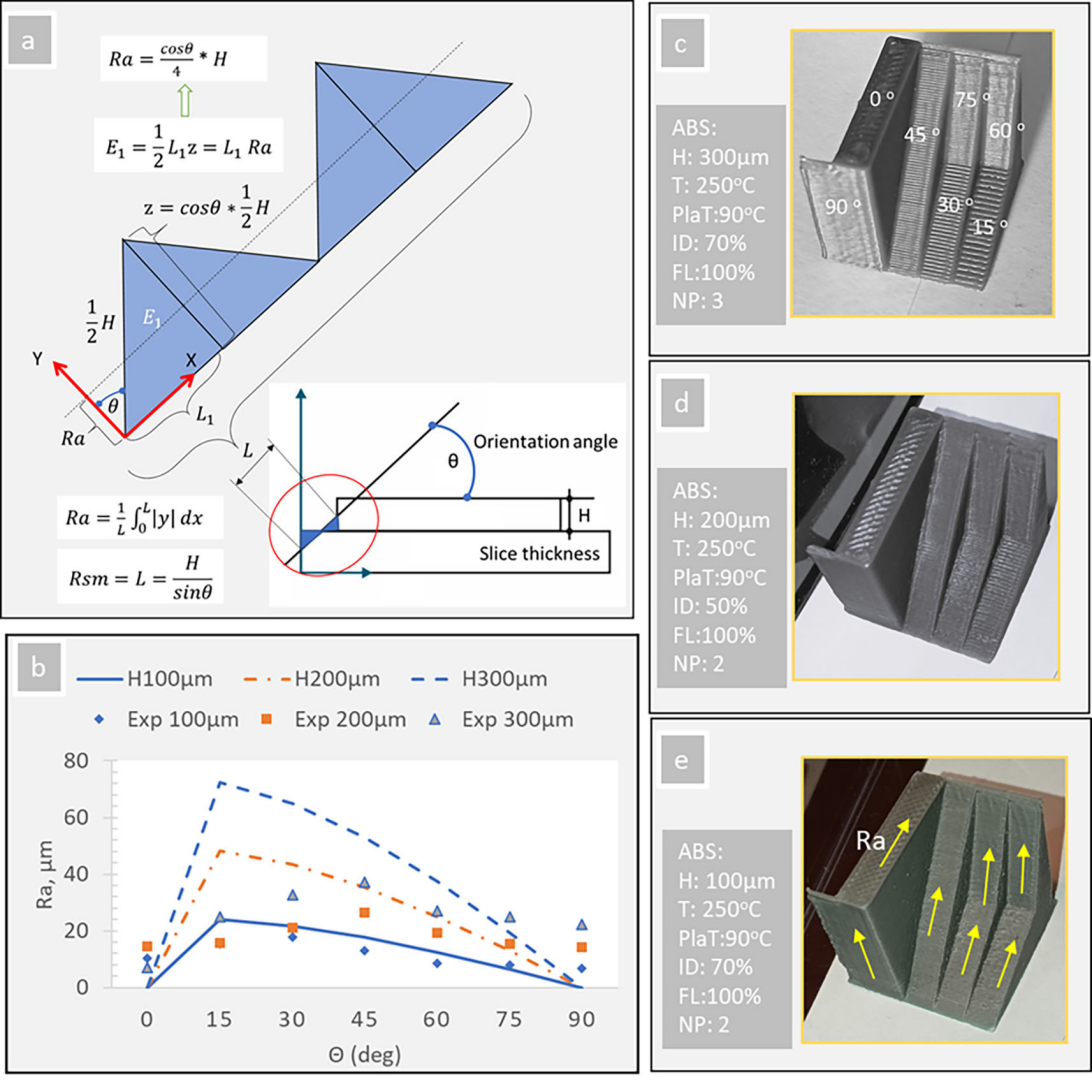
Stair-stepping effect on surface roughness (ABS material): (a) mathematical formula explanation and (b) graphical values with a prototype, and (c, d, e) experimental measurements (PlaT: Platform Temperature; NP: Perimeters number; FL: Flow rate; ID infill density; T: Nozzle temperature). **Source:** Author’s work.

Strand height (H) and surface orientation angle (θ) are the two directly influencing parameters (see Figure 7). In case the deposited material is assumed to have rectangular edges, the Ra and Rsm values are calculated with the following formulas (see explanation in Figure 7a):

$$\mathrm{Ra}=\frac{1}{L}\int_{0}^{L}|y|\mathrm{dx}=\frac{\text{cosΘ}}{4}H$$ (1)

$$\mathrm{Rsm}=L=\frac{H}{\text{sinθ}}$$ (2)

where Ra is the arithmetic mean height of the absolute values, Rsm is the mean value of the width of the profile elements, H is the strand (slice) thickness and θ is the surface orientation angle.

According to Eq. 1, higher H values and orientation angles between 5-30 degrees result in higher Ra values ( Figure 7b). Figure 7b shows the theoretical ( Eq.1) trend lines for 100, 200 and 300 μm H concerning the orientation angle (θ) and the corresponding experimental values (exp 100, 200 and 300μm). Figures 7c-e show the specially designed ABS-FFF test part and the oriented surfaces on which the average roughness (Ra) has been measured experimentally. The observed values follow the trend line only for 100μm (0.1 mm) H. More; lower Ra values were observed on the bottom surfaces, while the top surface texture appears to have significant Ra values. Finally, for 200 and 300μm H, the highest experimental Ra values were observed for the orientation angle of 45 degrees. Similar results have been marked by Boschetto *et al.,* ^80^ who applied artificial neural networks to predict surface roughness parameters (Ra, Rt, etc.).

### Overflow and layer trace

**Figure 8.**
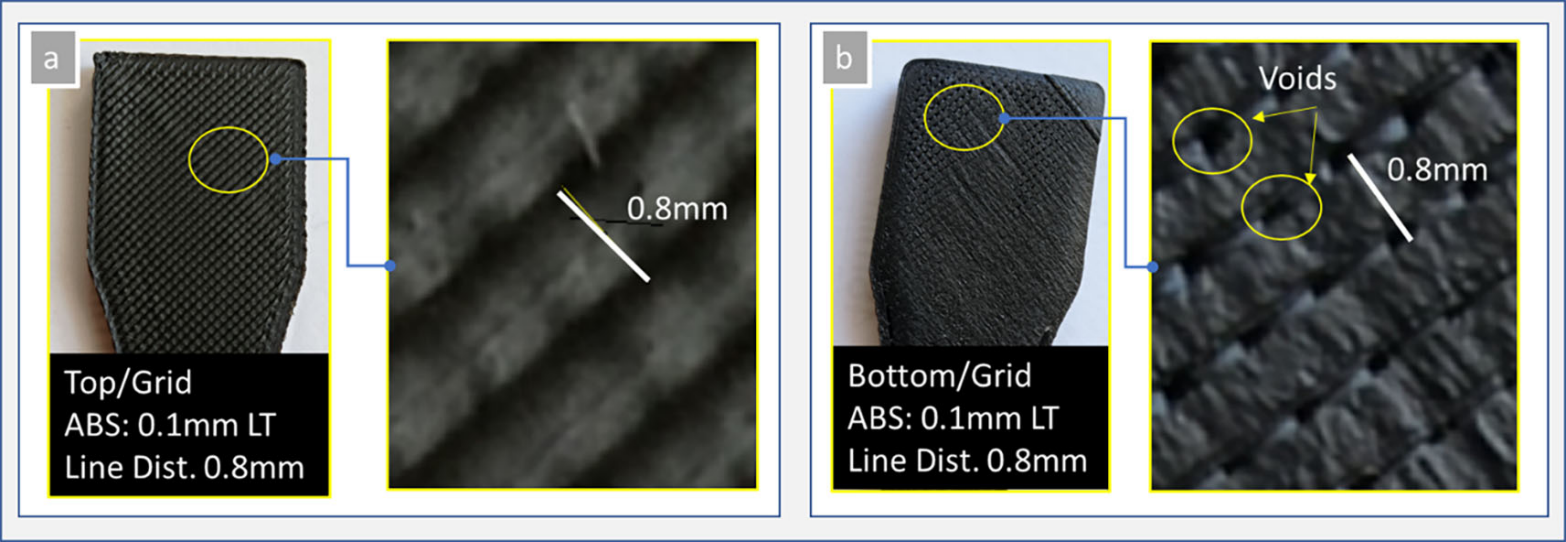
Welding conditions of top and bottom surfaces with grid style (0.4mm strand width and 0.8mm line distance, 250 ^o^C NT, 90 ^o^C platform temperature, 70% infill density, 100% flow rate): (a) top surface, and (b) bottom surface with grid style. **Source:** Author’s work.

The overflow effect is observed in all types of welding flat surfaces as well as on vertical surfaces. Top and bottom overflow is presented in Figure 5, where it is affected by the deposition conditions, i.e., the strand width, line distance, flow rate, nozzle inner and outer diameter, strand height, printing temperature, and printing speed. It is also possible to select zero top and bottom layers. In this case, the top and bottom layers follow the infill pattern grid style (see Figure 8). Therefore, the infill parameters must be adjusted appropriately so that voids are not observed (solid parts, 100% infill rate).

**Figure 9.**
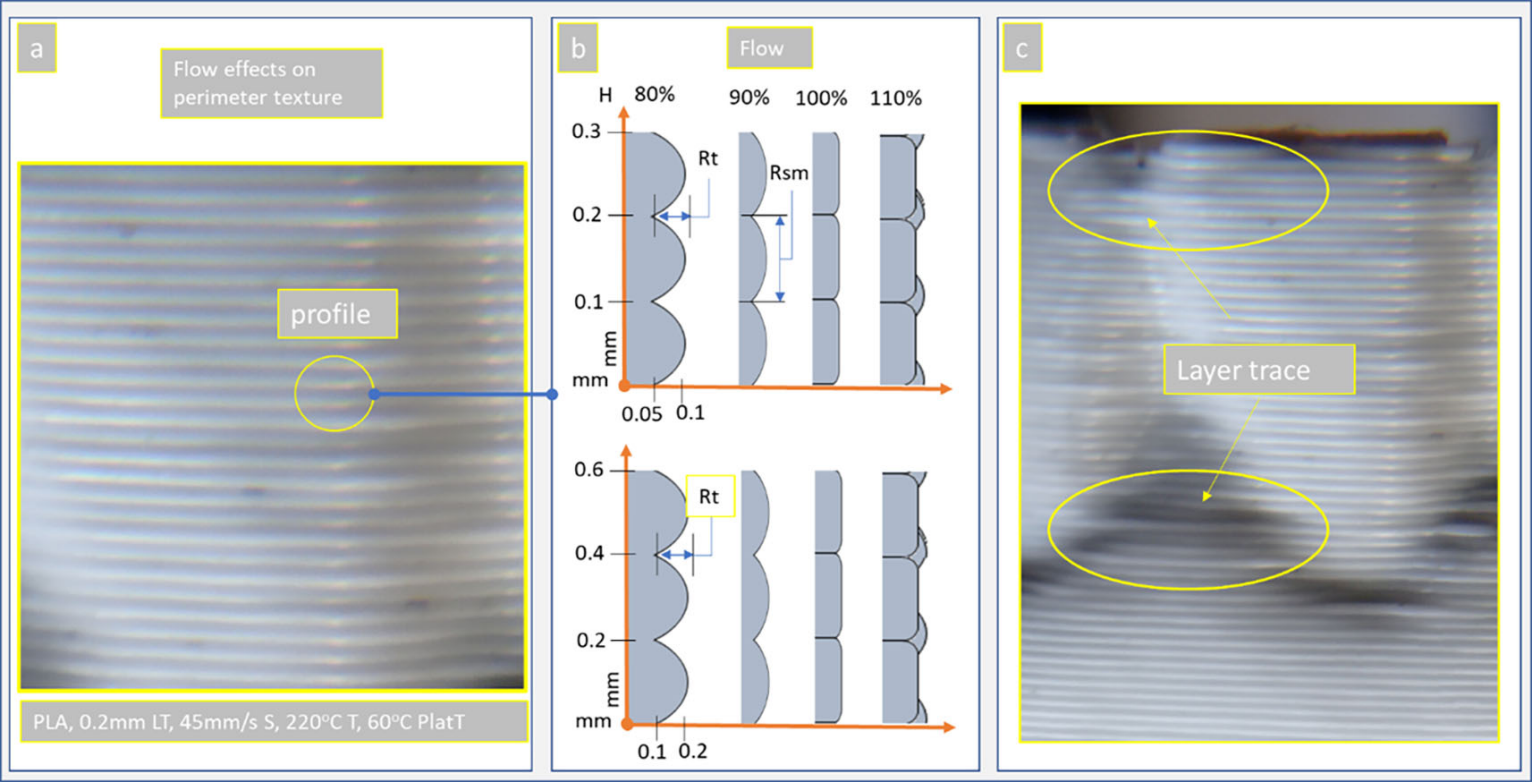
Void height (Rt) of the vertical surfaces regarding the material flow: (a) vertical surface texture, ^94^ (b) profile height for 0.1 and 0.2mm strand height, and (c) layer trace effect due to the deposition instability. **Source:** Author’s work.

On vertical or inclined surfaces, overflow occurs at the edges or start and end points of a line when the previous or next line changes direction due to the acceleration or deceleration of the nozzle at those locations (see Figure 6). ^142^ When the nozzle takes the printing velocity (S) the vertical and inclined surfaces have the profile of Figure 9a. This profile has gaps with max distance related of the layer height (H), material properties, and the flow rate (see Figure 9b). The gap height is about the same as the surface parameter Rt (difference between the highest peak and the deepest valley) and mean weave width about the Rsm surface parameter (see Eq. 2; the mean value of the width of the profile elements) which represents the layer height H for vertical surfaces (see Figure 9b). ^83^

Finally in Figure 9c the case of the layer trace effect due to the deposition instability of strands is highlighted. ^143^

### Filament properties

The ME process material can be a pure thermoplastic or a composite with a pure base material and a filler. ^144^ The filler could be carbon fibre, ^145^ graphene oxide, ^146^^,^^147^ glass fibers, ^148^ carbon nanotubes, ^149^ carbon black, ^150^ or organic powder, ^151^^,^^152^ to name a few. ^153^ Thermoplastic materials are divided into amorphous (ABS, ^154^^,^^155^ ABS/HDPE, ^156^ HIPS, ^157^ PET, ^158^ TPU, ^159^^,^^160^ etc.) and semi-crystalline (PLA, ^161^^,^^162^ PA12, ^163^ PEEK, ^164^ etc.).

**Figure 10.**
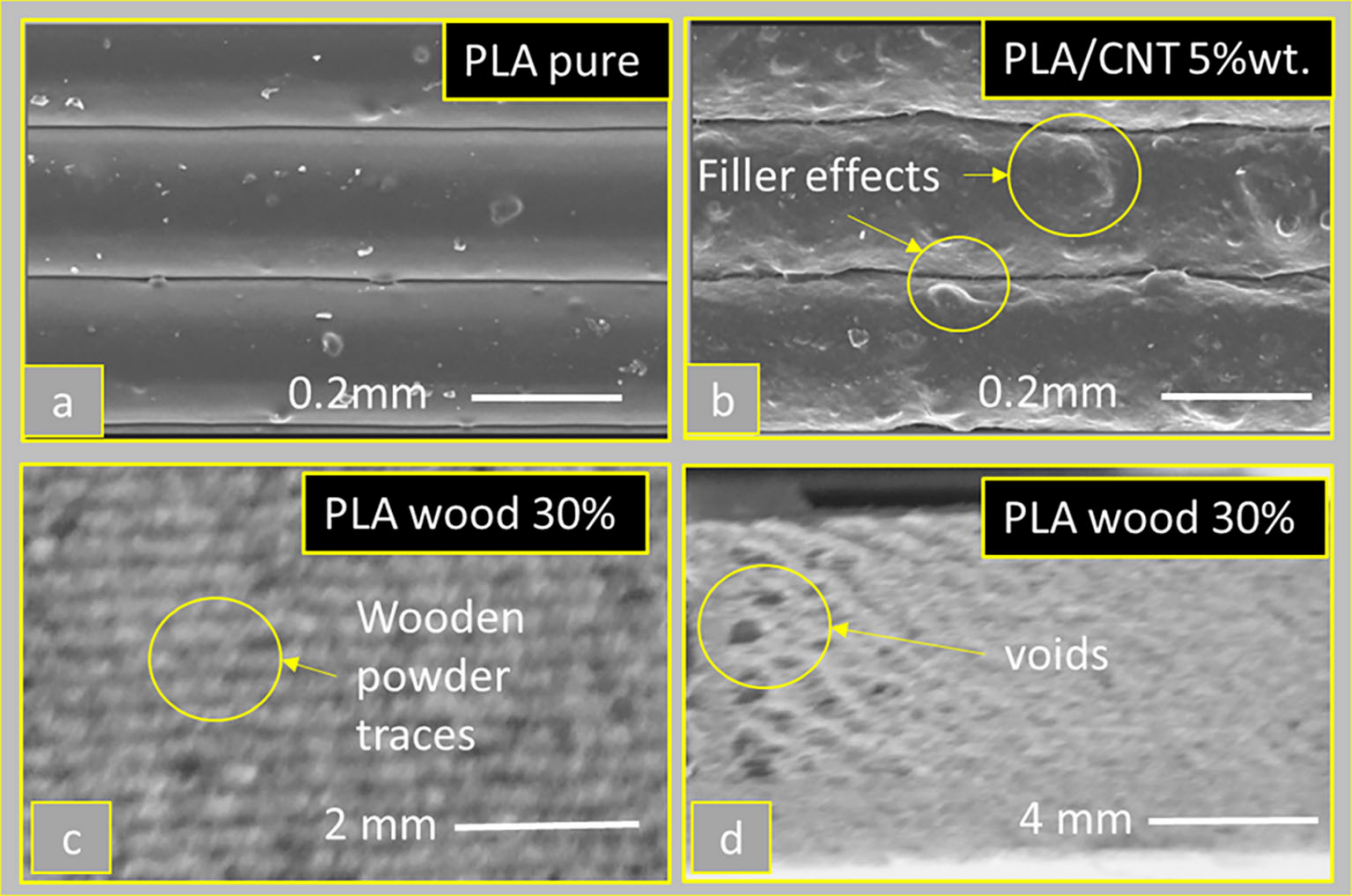
Filament material influence on the surface texture of deposited strands: (a) Pure PLA strands, (b) PLA with carbon nanotubes 5wt.%, ^69^ and (c, d) PLA with organic wood powders. **Source:** Author’s work.

The material’s properties determine the welding conditions more as the nozzle, bed and vat temperatures are adjusted accordingly. For example, filament strength and indentation shear through feed wheels play a critical role in material flow conditions and stability issues during the material deposition. In addition, the filler material directly affects the texture of ME 3D printing parts. Figure 10 shows the effects of filament material on vertical surfaces. For example, in Figure 10a (pure PLA), the substrates are smooth with a uniform texture, while in Figure 10b, the filler affects the surface texture by causing defects such as pillows and voids (5 wt.% CNT filler). Further, in Figures 10c and 10d, the effects of wood powder filler on pure PLA are shown with similar defects on the vertical surfaces.

## Discussion

The surface texture of the ME part is most affected by the material properties, the orientation of the part in the vat and the welding conditions during strands deposition. This is evident due to the process’s thermomechanical state and the material’s selective deposition following the g-codes positioning approach. Therefore, ME process performance is affected by some common defects, i.e., underflow or overflow, underheating or overheating, layer shifting, gaps between layers-perimeters-strands, curling and irregular corners resulting from welding processing parameters’ unsuitable setting. ^14^ As the ME process becomes more popular and broadens the choice of materials, surface roughness issues could become even more critical in future surface engineering applications, including metals and composites. ^165^ Note that the surface texture is crucial for the component’s durability and functionality in parts’ assemblies and systems such as external and internal thread, ^166^ helical gears, ^167^ remanufacturing of damaged or broken parts cases, ^168^ custom-made anatomical orthotics, ^169^ etc. Thus, many materials that can be extruded in filament form should be tested and investigated for surface texture and geometric quality characteristics, ^170^ roughness, ^95^ conductivity, ^171^ hardness, ^94^ friction properties, ^172^^,^^173^ tribological and wear properties, ^174^ and aesthetics, ^175^ of 3D printed parts in all directions, not just on flat surfaces of mechanical test specimens. ^176^

Additionally, intent shear effects of the filament during nozzle feeding, nozzle outer vs inner diameter associated with each material composition, the appropriate flow rate for each nozzle type vs material selection combination, and optimization of welding parameters are just some of the future challenges of the ME process on the surface texture quality issues of 3D printed parts. In addition, stability issues that will improve nozzle effects on layer traces, material overflow effect in edges, and texture weaving approaches could further enhance the surface quality of ME 3D printed parts. ^177^

Considering all the above issues, the surface texture of the ME part can be significantly improved uniformly in all the mentioned cases below 10-15μm, reducing the time and cost of further post-processing by blasting, ^136^ chemical treatment, ^96^^,^^170^ or machining. ^97^ For example, Francis *et al.,* ^96^ improved surface texture to 0.819μm from 9μm with 50s immersion time in acetone solvent.

Last but not least, the effect of the support parameters on the surface roughness of the top and bottom surfaces is another challenge for the ME process (see Figure 2). Therefore, it should be studied concerning the characteristics and integrity of the ME parts.

## Conclusions

In the ME process, many parameters affect the surface texture and properties of 3D-printed parts. In this study, the types of surface texture encountered in the ME process were first analysed. e.g., top, bottom, vertical and sloped and after studying the effects of the stair-stepping, overflow and filament material. This study presents for the first time in detail the complex process of surface texture control in the ME 3D-printing process as, for each case, some processing parameters have competing effects and others synergistic effects.

• Surface orientation (θ) and layer height defined by the strand height (H) are significant for the stair-stepping effect.
• Material filament fillers negatively affect all surfaces, increasing the Ra and Rt values. Today, new materials are being investigated, such as metal powders on polymer bases. As a result, researchers are constantly expanding the materials used in ME.
• Strand width (w), printing speed (S) and flow rate affect the overflow effect and are significant for vertical, inclined and flat surfaces.
• Surface texture properties can be significantly improved by 3D printing parameters optimization.
• Post-processing time and cost can consequently be reduced considerably.

Improving the ME surface texture properties will speed up the ME technology in more surface technology applications, such as mechanical applications in systems and assemblies or remanufacturing cases.

## Data Availability

All data generated or analyzed during this study are included in the manuscript.
