# Supplementary material for: Effects of thermomechanical parameters on surface texture in filament materials extrusion: outlook and trends
Source: F1000Res. 2024 Sep 10;13:1039. [Version 1] doi: 10.12688/f1000research.144965.1 (PMC11541081; doi:10.12688/f1000research.144965.1)
Supplement: Peer review history

# Reviewer report: Reviewer response for version 1

**DOI:** 10.5256/f1000research.158837.r333470

**Published:** 2024-11-06

**Authors:** Muhammad Abas

**Affiliations:** 1 University of Engineering and Technology,, Peshawar, Pakistan

The article is well presented and provide in depth information about the surface texture in material extrusion. However, the few comments need be addressed before final acceptance.

 1. Please include some recent references (2023-2024). https://doi.org/10.1108/RPJ-10-2023- (ref-1)0347, 

https://doi.org/10.3390/polym15173633 (ref-2), 

https://doi.org/10.1016/j.addma.2024.103960 (ref-3)

 2. Please include a discussion on standard procedures for measuring surface roughness, such as those based on ISO standards.

Is the review written in accessible language?

Yes

Are all factual statements correct and adequately supported by citations?

Yes

Are the conclusions drawn appropriate in the context of the current research literature?

Yes

Is the topic of the review discussed comprehensively in the context of the current literature?

Yes

Reviewer Expertise:

Additive Manufacturing

I confirm that I have read this submission and believe that I have an appropriate level of expertise to confirm that it is of an acceptable scientific standard.

## References

[1] : 3D printing of personalised stents using new advanced photopolymerizable resins and Ti-6Al-4V alloy. *Rapid Prototyping Journal* .2024;30(4) : 10.1108/RPJ-10-2023-0360 696-710 10.1108/RPJ-10-2023-0360

[2] : Analyzing Surface Roughness Variations in Material Extrusion Additive Manufacturing of Nylon Carbon Fiber Composites. *Polymers (Basel)* .2023;15(17) : 10.3390/polym15173633 10.3390/polym15173633 37688259 PMC10489770

[3] : Effects of in-process surface finishing on part strength in polymer material extrusion additive manufacturing. *Additive Manufacturing* .2024;80: 10.1016/j.addma.2024.103960 10.1016/j.addma.2024.103960

# Reviewer report: Reviewer response for version 1

**DOI:** 10.5256/f1000research.158837.r324925

**Published:** 2024-10-03

**Authors:** Vivek Kumar Tiwary

**Affiliations:** 1 KLS Gogte Institute of Technology, Belgaum, Karnataka, India

The research paper is good and can be indexed. It is very relevant to the topic of Material Extrusion. The paper comprehensively examines material extrusion process achievements in surface texture quality and studies and summarizes the most influential processing parameters. Parameter effects are critically discussed for each topic; flat, inclined, and vertical surfaces. The results of this research will help the scientific community help reduce post-processing requirements on 3D printed components.

Is the review written in accessible language?

Yes

Are all factual statements correct and adequately supported by citations?

Yes

Are the conclusions drawn appropriate in the context of the current research literature?

Yes

Is the topic of the review discussed comprehensively in the context of the current literature?

Yes

Reviewer Expertise:

FDM 3D Printing

I confirm that I have read this submission and believe that I have an appropriate level of expertise to confirm that it is of an acceptable scientific standard.
